# Charge-Stripe Order and Superconductivity in Ir_1−x_Pt_x_Te_2_

**DOI:** 10.1038/s41598-017-16945-7

**Published:** 2017-12-07

**Authors:** O. Ivashko, L. Yang, D. Destraz, E. Martino, Y. Chen, C. Y. Guo, H. Q. Yuan, A. Pisoni, P. Matus, S. Pyon, K. Kudo, M. Nohara, L. Forró, H. M. Rønnow, M. Hücker, M. v. Zimmermann, J. Chang

**Affiliations:** 1 Physik-Institut, Universität Zürich, Winterthurerstrasse 190, CH-8057, Zürich, Switzerland; 2 Laboratory of Physics of Complex Matter, Institute of Physics, Ecole Polytechnique Féderale de Lausanne (EPFL), CH-1015 Lausanne, Switzerland; 3 Laboratory for Quantum Magnetism, Institute of Physics, Ecole Polytechnique Féderale de Lausanne (EPFL), CH-1015 Lausanne, Switzerland; 4 Center for Correlated Matter and Department of Physics, Zhejiang University, 310027 Hangzhou Zhejiang, People’s Republic of China; 5 Department of Applied Physics, The University of Tokyo, Tokyo, 113-8656 Japan; 6 Research Institute for Interdisciplinary Science, Okayama University, Okayama, 700-8530 Japan; 7 Department of Condensed Matter Physics, Weizmann Institute of Science, Rehovot, 7610001 Israel; 8 Deutsches Elektronen-Synchrotron DESY, 22603 Hamburg, Germany

## Abstract

A combined resistivity and hard x-ray diffraction study of superconductivity and charge ordering in Ir Ir_1−x_Pt_x_Te_2_, as a function of Pt substitution and externally applied hydrostatic pressure, is presented. Experiments are focused on samples near the critical composition *x*_*c*_ ~ 0.045 where competition and switching between charge order and superconductivity is established. We show that charge order as a function of pressure in Ir_0.95_Pt_0.05_Te_2_ is preempted — and hence triggered — by a structural transition. Charge ordering appears uniaxially along the short crystallographic (1, 0, 1) domain axis with a (1/5, 0, 1/5) modulation. Based on these results we draw a charge-order phase diagram and discuss the relation between stripe ordering and superconductivity.

## Introduction

Transition-metal dichalcogenides have long been the centre of considerable attention because of their complex quasi two-dimensional electronic properties. Semiconductor physics^1^, superconductivity^2–4^ and spontaneous breaking of lattice symmetry, driven by charge-density waves (CDW)^5–7^, are commonly reported. Often, the ground state properties of these materials can be controlled by external non-thermal parameters such as chemical substitution^8^, magnetic field^9,10^ or hydrostatic pressure^11^. The prototypical 1T-TaS_2_ compound can, for example, be tuned from a CDW state to superconductivity by application of hydrostatic pressure^11^. Recently, a connection between charge density wave order in 1T-TaS_2_ and orbital textures has been demonstrated^12^. A parallel effort has been to study dichalcogenide systems in which spin-orbit coupling is considerable. To this end, IrTe_2_ has attracted interest because spin-orbit coupling on the Ir site is known to be large^13,14^. The IrTe_2_ system displays high-temperature charge ordering, and superconductivity can be induced by Pt or Pd substitution that in turn quenches the charge order^15–17^. Several studies concluded in favour of a conventional *s*-wave pairing symmetry^18,19^. It remains however to be understood how charge order, lattice symmetry and superconductivity interfere.

In the parent compound IrTe_2_, charge order coincides with a lowering of the crystal structure symmetry (from hexagonal P$\overline{3}m1$ to monoclinic C2/*m*)^15^. This effect is most likely not accidental and hence IrTe_2_ falls into the category of materials such as La_2−*x*_Ba_*x*_CuO_4_^20^, Ca_2_RuO_4_^21,22^, and URu_2_Si_2_^23^ where structural and electronic transitions appear simultaneously. For such systems, it is important to address the question whether the transition is lattice or electron driven. Resolving this issue, is often crucial to understand the electronic instability. The fact that superconductivity emerges when charge order is quenched by chemical pressure tuning, is probably also not coincidental. It may indicate that quantum criticality enters as a supporting ingredient to the formation of superconductivity. The interplay between charge ordering and superconductivity is therefore an interesting topic to explore. Charge ordering of the parent compound has been studied in great detail, and it has been shown how different modulation vectors emerge as a function of temperature. Upon cooling the system first develops a (1/5, 0, 1/5) modulation (*T* < 280 K) that switches to (1/8, 0, 1/8) at lower temperatures^24–26^, (*T* < 200 K). There exist, however, no x-ray diffraction studies of the charge order in Ir_1−x_Pt_x_Te_2_ near the critical composition (*x*_*c*_ ~ 0.045) for superconductivity.

Here we present a combined resistivity and x-ray diffraction study of Ir_1−x_Pt_x_Te_2_ as a function of chemical substitution and hydrostatic pressure near the critical composition *x*_*c*_. Just below this critical composition, we find a temperature independent charge ordering modulation vector (1/5, 0, 1/5). This signifies a difference from the parent compound where the ground state charge modulation is (1/8, 0, 1/8)^25,26^. Our pressure experiments were carried out just above *x*_*c*_ (namely at *x* = 0.05) in a compound with a superconducting ground state and no evidence of charge order at, and around, ambient conditions 1–400 bar. With increasing pressure, we find a lowering of lattice symmetry above *p*_*c*1_ ~ 11.5 kbar. This breaking of the hexagonal lattice symmetry appears without any trace of charge ordering that emerges only for pressures above *p*_*c*2_ ~ 16 kbar. From this observation we conclude that charge ordering is lattice – rather than electronically – driven. Combining our results with those previously obtained in IrTe_2_, we propose a charge order phase diagram as a function of Pt substitution and hydrostatic pressure. In terms of structure, we demonstrate that charge ordering is appearing unidirectionally along the short lattice parameter axis. Finally, we discuss the interplay between charge ordering and superconductivity. The temperature versus Pt substitution phase diagram^15^ suggests that these two phases are competing. Based on our resistivity data, we argue that superconductivity may survive into the uniaxial charge ordering phase however the transition gradually broadens to a point where zero resistance is not observed. We discuss possible explanations of this effect in terms of (1) chemical and electronic inhomogeneity, (2) granular superconductivity and (3) a three- to two-dimensional electronic transition.

## Results

**Figure 1:**
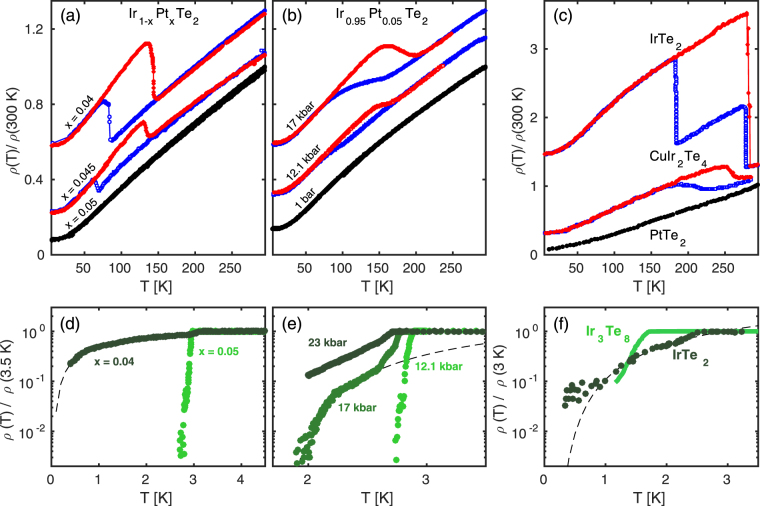
Warming and cooling resistivity curves for Ir_1–*x*_Pt_*x*_Te_2_ and related stoichiometric compounds. (**a**) Substitution dependence for Pt concentrations as indicated. (**b**) Resistivity measured on Ir_0.95_Pt_0.05_Te_2_ and hydrostatic pressures as indicated. (**c**) Resistivity curves for the parent compound IrTe_2_, and related materials CuIr_2_Te_4_ and PtTe_2_ (adapted from refs^26,48^). For the sake of visibility, the colored curves in (**a**,**b**) and (**c**) have been given an arbitrary shift. (**d**) and (**e**) display the low-temperature resistivity curves recorded under the same conditions as in (**a**) and (**b**). (**f**) Comparable resistivity curves of the stoichiometric compounds IrTe_2_ and Ir_3_Te_8_ adapted from refs^39,49^. Dashed lines in (**d**)–(**f**) are guides to the eye only.

Cooling and warming resistivity curves are plotted in Fig. 1, for different compositions of Ir_1−x_Pt_x_Te_2_ as indicated. Similar curves are shown for Ir_0.95_Pt_0.05_Te_2_ for different levels of hydrostatic pressures as indicated. The hysteresis loops indicate a first order transition that certainly is related to the lowering of crystal lattice symmetry and/or the emergence of charge order. From the resistivity curves, alone, it is however not possible to determine whether the transition is electronic or lattice driven. To illustrate this point, we show in Fig. 1(c) resistivity curves of the stoichiometric compounds IrTe_2_, CuIr_2_Te_4_ and PtTe_2_. Among these materials, charge ordering has only been observed in IrTe_2_. The hysteretic resistive behaviour of CuIr_2_Te_4_ is therefore not caused by charge ordering, but rather by a structural transition. In Fig. 1(d) and (e) the superconducting transition of Ir_1−x_Pt_x_Te_2_ is displayed and compared to the stoichiometric compounds IrTe_2_ and Ir_3_Te_8_ [Fig. 1(f)]. Empirically, it seems that the superconducting transition broadens dramatically in the coexistent regime.

**Figure 2:**
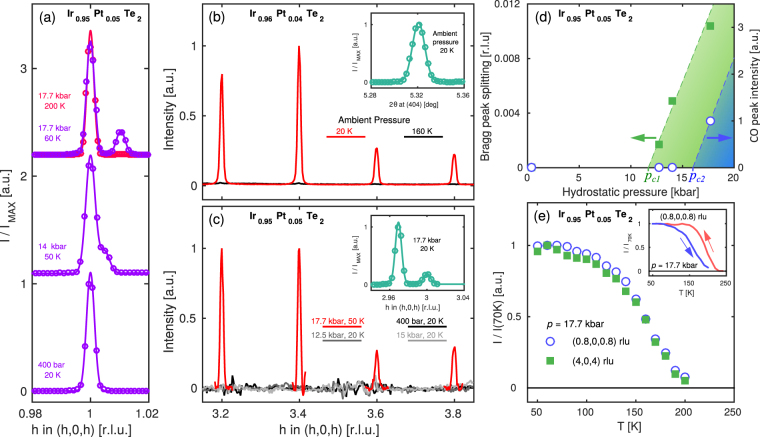
Lattice and charge ordering reflections in Ir_1 – x_Pt_x_Te_2_. (**a**) Bragg peak (1, 0, 1) reflection measured in Ir_0.95_Pt_0.05_Te_2_ as a function of pressure as indicated. Solid lines are Gaussian fits to the data. (**b**) Ambient pressure x-ray diffracted intensity measured on Ir_0.96_Pt_0.04_Te_2_ along the (1, 0, 1) direction for 20 K (red line) and 160 K (black line) respectively. (**c**) Scan as in (**b**) but measured at base temperature (20 K) on Ir_0.95_Pt_0.05_Te_2_ for pressures as indicated. The slightly worse signal-to-noise level stems from the necessary background subtraction of signal originating from the pressure cell. (**d**) Bragg peak splitting and charge ordering intensity – shown in (**a**) and (**c**) – as a function of pressure. (**e**) Temperature dependence of the intensity of charge ordering and short-axis reflections on Ir_0.95_Pt_0.05_Te_2_ with maximum applied pressure, as indicated. Warming and cooling intensities of charge ordering are shown in the inset.

To gain further insight into the relation between the lattice and charge order, we carried out an x-ray diffraction study. In Fig. 2(a), we show the fundamental lattice Bragg peak τ = (1, 0, 1) measured at low temperature on Ir_0.95_Pt_0.05_Te_2_ at different pressures as indicated. At low pressure (*p* = 400 bar) a single sharp Bragg peak is observed. Above a critical pressure *p*_*c*1_, this peak develops a shoulder that upon further increased pressure evolves into a separate Bragg peak. When heating above 200 K, this Bragg peak splitting disappears. Altogether, this evidences a low-temperature pressure-induced lowering of the lattice symmetry.

Next, we explore the charge ordering. **Q**-scans recorded on Ir_0.96_Pt_0.04_Te_2_ along the (*h*, *0*, *h*) high symmetry direction are displayed in Fig. 2(b). Just as reported in IrTe_2_^24,25^, no twinning was observed on Bragg peaks equivalent to **τ** = (1, 0, 1) – see inset. Moreover, below 160 K strong charge order reflections are observed at wave vectors **Q** = **τ** + **q**_*co*_ where **q**_*co*_ = (±1/5, 0, ±1/5) and (±2/5, 0, ±2/5) and **τ** are fundamental Bragg reflections. We find (not shown) that off-diagonal reflections of the type (*h*, *0*, *h* + *n*) with *n* = 1, 2, 3 are much weaker than for *n* = 0. As the diffracted intensity *I* is proportional to **Q · u** where **u** is the atomic displacement^27,28^, we conclude that displacements are predominately along the (*h*, *0*, *h*) direction.

With this knowledge, we studied the charge order in the pressure-induced twinned phase of Ir_0.95_Pt_0.05_Te_2_. The crystal was carefully aligned on the **τ** = (3, 0, 3) Bragg peak using the larger lattice constant. At the highest applied pressure $p\simeq 17.7$ kbar, a **q**_*co*_ = (±1/5, 0, ±1/5) charge modulation is observed with respect to the Bragg peak with the shorter lattice parameter [see Fig. 2(c)]. The charge ordering reflection displays, just as the resistivity curves, hysteretic behaviour as a function of temperature [inset of Fig. 2(e)]. Finally, we show in Fig. 2(e) how upon cooling the charge order reflection and the short-axis Bragg peak **τ** = (4, 0, 4) have identical temperature dependence. This demonstrates an intimate relation between the crystal lattice symmetry breaking and charge ordering.

## Discussion/Interpretation

### Lattice vs electronic mechanism

**Figure 3:**
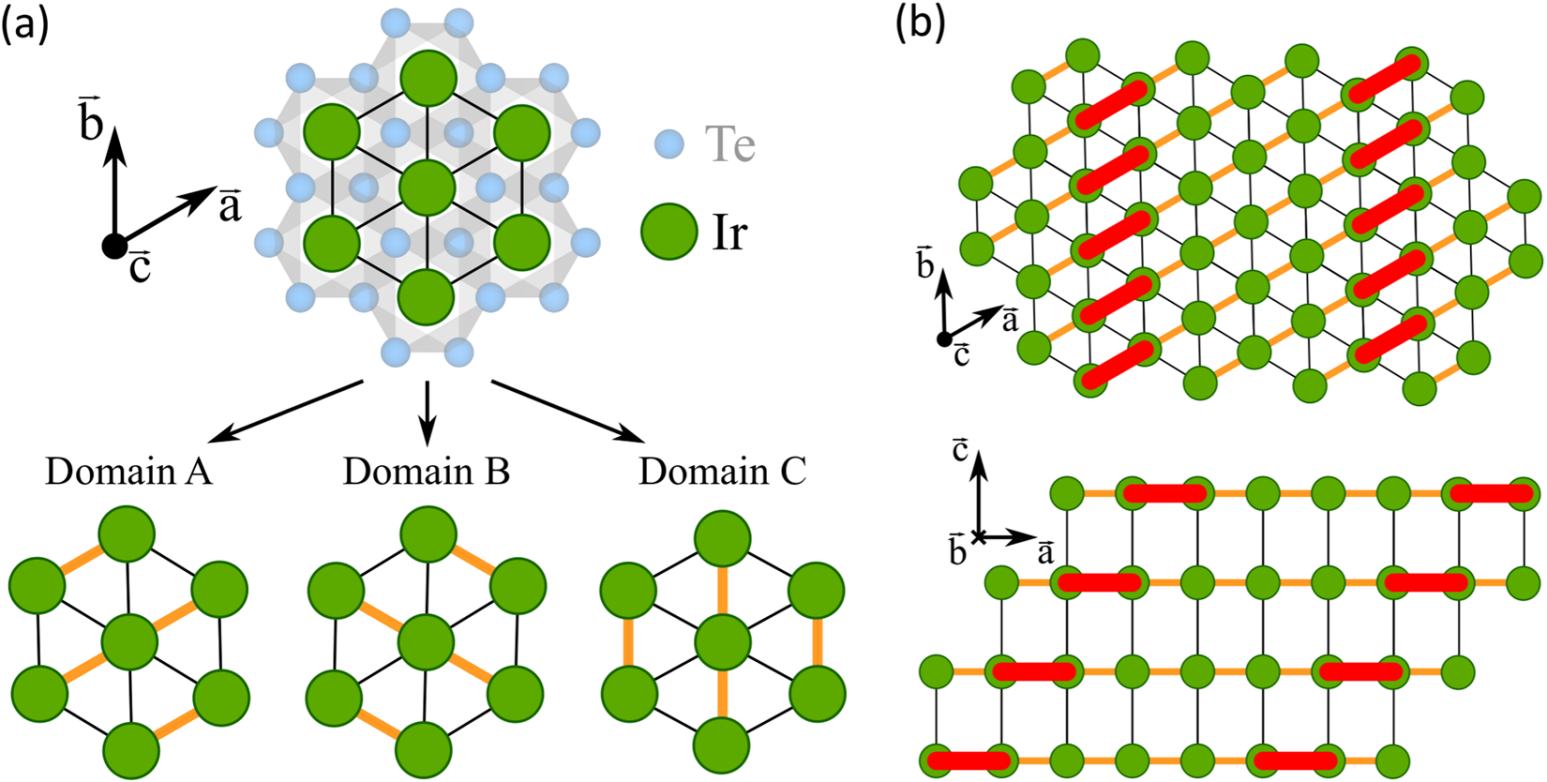
(**a**) Projection of the hexagonal crystal structure of IrTe_2_. The transition into monoclinic structure implies formation of three domains where a short lattice parameter axis is found along the $\overrightarrow{a}$, $\overrightarrow{b}$ or $\overrightarrow{a}-\overrightarrow{b}$ direction. These domains are labeled A, B and C respectively. (**b**) Stripe charge order forms along the short axis direction. The Ir^3+^- Ir^3+^ dimers – indicated by red bonds – intersect the crystal structures with $\overrightarrow{b}$, $\overrightarrow{a}+\overrightarrow{c}$ planes.

**Figure 4:**
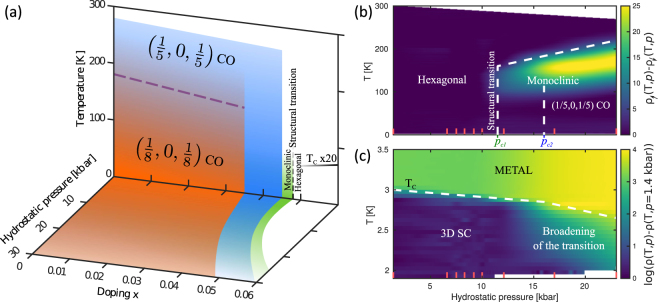
(**a**) Schematic pressure - temperature phase diagrams of the charge ordering and crystal lattice twinning of Ir_1−x_Pt_x_Te_2_. (**b**) Hydrostatic pressure vs temperature map of the difference between the warming and cooling resistivity curves of Ir_0.95_Pt_0.05_Te_2_ represented in false colours. (**c**) Similar map but for the difference of each resistivity curve with the one measured at 1.4 kbar in the superconductor transition temperature range (displayed in logarithmic-intensity scale). Red ticks indicate the measured pressures. White dashed lines are guides to the eye.

We start by discussing the nature of the charge ordering transition. The pressure-induced Bragg peak splitting [Fig. 2(a)] is most naturally explained in terms of domain formation caused by a lowering of the crystal lattice symmetry. In essence, our experiment suggests that the lattice parameters along the (1, 0, 1) and (0, 1, 1) directions become inequivalent under application of pressure. The system thus develops three domains with a short lattice parameter along the $\overrightarrow{a}$, $\overrightarrow{b}$ or $\overrightarrow{a}-\overrightarrow{b}$ axes, see Fig. 3(a). All three types of domain are observed when scanning along the (1, 0, 1) direction in the pressure-induced twinned phase and hence two Bragg peaks are found – shown in Fig. 2(a). This twinning effect clearly appears before charge ordering, suggesting that the latter is lattice driven. Given that we observe the same (1/5, 0, 1/5) modulation as in IrTe_2_ (high-temperature), it is not inconceivable that the same conclusion applies to the parent compound. Combining our results with previous studies of IrTe_2_, we propose in Fig. 4(a) a schematic pressure, Pt substitution and temperature phase diagram including the charge ordering and the structural hexagonal to monoclinic transition.

### Charge order structure

The surface and bulk charge ordering structure of IrTe_2_ has been studied by scanning tunnelling microscopy (STM)^29–34^ and x-ray diffraction^24,25,35^ techniques. The STM studies generally find uniaxial charge ordering structures. Furthermore, differences in charge modulations between the bulk and surface have been pointed out^34^. Our bulk-sensitive results on Ir_0.95_Pt_0.05_Te_2_ indicate that the pressure-induced charge order is connected to the short-axis direction only. Therefore, the most simple explanation is uniaxial Ir^3+^- Ir^3+^ dimer formation along the short lattice parameter axis as illustrated in Fig. 3(b). For such a structure, an electronic gap is expected only along the reciprocal short lattice parameter axis. However since the crystals are inevitably twinned along three different directions, it can be challenging to observe with angle resolved photoemission spectroscopy (ARPES) experiments, in particular when factoring in the complex electronic band structure^36–38^. A suppression of the spectral weight (near the Fermi level) is observed with ARPES and optical experiments. This observation is at odd with a conventional charge density wave and hence taken as evidence of novel type of charge ordering^36,38,39^.

### Superconductivity and Charge order

Finally, we discuss the relation between unidirectional charge order and superconductivity. From our pressure-dependent x-ray and resistivity experiments, we show that a lowering of the crystal symmetry has no impact on superconductivity [see Figs 1(e) and 4(b)]. Upon entering into the charge ordered phase, the superconducting transition however, broadens dramatically. While the initial superconducting onset remains fairly constant, the onset of zero resistance (within the detection limit) undergoes dramatic changes. In fact as a function of pressure, the system quickly reaches a regime where zero resistance is not observed within the measured temperature window [see Fig. 1(e)]. The same trend is found at ambient pressure when lowering the Pt content [see Fig. 1(d) and (f)]. Hence there seems to be a correlation between the occurrence of the charge order and a broadening of the superconducting transition. On general grounds, such a broadening can have different explanations. (1) Chemical or electronic inhomogeneities can smear the transition. (2) Granular superconductivity is also characterised by broad transitions. (3) Low-dimensional superconductivity is known to introduce two temperature scales. In particular, for two-dimensional superconductivity, an exponential resistive drop, approximately described by $\rho (T)\propto \exp (\frac{-b}{\sqrt{t}})$, is expected below *T*_*c*_. Here *b* is a constant and $t=(T-{T}_{c}^{3D})/{T}_{c}^{3D}$ with ${T}_{c}^{3D}$ being a second superconducting temperature scale. This Kosterlitz - Thouless transition^40,41^, scenario finds its relevance in Ir_1−x_Pt_x_Te_2_, since charge order is shown to generate two dimensional walls of low density-of-states^24,25,42–44^. It is therefore not inconceivable that superconductivity is suppressed inside these walls. Hence there exists a possible physical mechanism for two-dimensional superconductivity in Ir_1−x_Pt_x_Te_2_. Further experimental evidence supporting this scenario would be of great interest. Based on the experimental evidence presented here, it is difficult to prove the Kosterlitz - Thouless scenario. Nor can we completely exclude inhomogeneities or grain boundaries. Chemical inhomogeneity is very unlikely to be the cause, since it should not be influenced by hydrostatic pressure. Inhomogeneous pressure can also be excluded as the broadening is found also at ambient pressure [see Fig. 1(d)]. Intrinsic electronic inhomogeneity could be tuned by both pressure and chemical substitution. However, one would expect that inhomogeneity generates more modest correlation length of the charge order. Experimentally, however, long range (resolution limited) charge order reflections are observed. The domain formation makes the granular superconducting scenario more plausible. We notice, however, that the pressure induced crystal domain formation initially have no influence on superconductivity. Explaining our data in terms of granular superconductivity is therefore not straightforward.

## Conclusion

In summary, we have presented a combined resistivity and x-ray diffraction study of Ir_1−x_Pt_x_Te_2_ as a function of Pt substitution and hydrostatic pressure. Just below the critical composition *x*_*c*_ ~ 0.045 charge order with a (1/5, 0, 1/5) wave vector is found. The same modulation appears in Ir_0.95_Pt_0.05_Te_2_ upon application of hydrostatic pressures beyond *p*_*c*2_ ~ 16 kbar. Based on these observations a charge ordering phase diagram is constructed. Application of pressure furthermore revealed a lattice symmetry lowering transition appearing before the charge ordering. We thus conclude that the charge ordering in Ir_1−x_Pt_x_Te_2_ is lattice driven. Finally, we discussed the relation between charge order and superconductivity.

## Methods

Single crystals of Ir_1−x_Pt_x_Te_2_ were grown using a self-flux technique^39^. Piston-type pressure cells^45^ with Daphne oil as pressure medium were used to reach ~18 kbar and 23 kbar, for x-ray diffraction and resistivity experiments respectively. The hydrostatic pressure was estimated from the orthorhombicity of La_1.85_Ba_0.125_CuO_4_ at 60 K^46^ and the resistive superconducting transition of lead. The electrical resistivity was measured by a conventional four-probe method using a physical property measurement system (Quantum Design PPMS-14T) and hard x-ray diffraction (100 keV) experiments were carried out with the triple-axis instrument at beamline P07 at PETRA III, DESY. Although Ir_1−x_Pt_x_Te_2_ at certain temperatures and pressures displays crystal structure twinning, the momentum **Q** = (*h*, *k*, *l*) is presented in hexagonal notation with *a* ≈ *b* ≈ 3.95 Å and *c* ≈ 5.38 Å. Crystallographic projections were produced using the VESTA software^47^.

The datasets generated during and/or analysed during the current study are available from the corresponding author on reasonable request.
